# Hemostasis of Left Atrial Appendage Bleed With Lariat Device

**DOI:** 10.1016/s0972-6292(16)30800-2

**Published:** 2014-10-06

**Authors:** Amena Hussain, Muhamed Saric, Scott Bernstein, Douglas Holmes, Larry Chinitz

**Affiliations:** NYU Langone Medical Center, United States

**Keywords:** Left Atrial Appendage, Lariat Device

## Abstract

New devices designed for minimally invasive closure of the left atrial appendage (LAA) may be a viable alternative for patients in whom anticoagulation is considered high risk. The Lariat (Sentreheart, Redwood City, CA), which is currently FDA-approved for percutaneous closure of tissue, requires both trans-septal puncture and epicardial access. However it requires no anticoagulation after the procedure. Here we describe a case of effusion and tamponade during a Lariat procedure with successful completion of the case and resolution of the effusion.

## Introduction

New devices designed for minimally invasive closure of the left atrial appendage (LAA) may be a viable alternative for patients in whom anticoagulation is considered high risk. The Lariat (Sentreheart, Redwood City, CA), which is currently FDA-approved for percutaneous closure of tissue, requires both trans-septal puncture and epicardial access. However it requires no anticoagulation after the procedure. Here we describe a case of effusion and tamponade during a Lariat procedure with successful completion of the case and resolution of the effusion.

## Case

An 87 year old man with longstanding persistent atrial fibrillation and elevated stroke risk (CHADSVASC score = 3, HASBLED = 2) was felt not to be candidate for chronic oral anticoagulation due to repeated falls associated with orthopedic injury. He was referred to our Electrophysiology Laboratory for percutaneous suture ligation of the left atrial appendage.

A pre-operative CT angiogram was performed to determine the patient's left atrial appendage anatomy. This showed a single lobed appendage 3.3cm in length with a trabeculated distal portion and was deemed suitable for the exclusion procedure. Under general anesthesia, intra-operative 3D transesophageal echocardiography (TEE) demonstrated no left atrial thrombus. A Tuohy needle was used to enter the pericardial space via a subxiphoid approach under fluoroscopic guidance. Entry into the pericardial space was anterior-lateral, using contrast and a lateral fluoroscopic view. The access site was sequentially dilated and a 13Fr sheath was placed into the pericardial space. Next, trans-septal puncture was performed using an 8.5 Fr SL-1 sheath and an Extra Sharp Brokenbrough needle, via the right femoral vein. A Heparin drip was initiated to maintain adequate activated clotting time (250-300 sec).

A left atrial angiogram with an occlusive balloon (SentreHeart Endocath) confirmed the LAA size, shape and orientation, consistent with CTA findings. A magnet-tipped guidewire (Sentreheart FindrWIRZ) wire was advanced into the distal portion of left atrial appendage. A second magnet-tipped guidewire was advanced into the subxiphoid space and coupled magnetically to the LAA wire, in the end-to-end configuration. The Lariat suture loop was then advanced into the pericardial space over the magnet guidewire. The suture loop could not be advanced to the base of the appendage due to separation of the guidewire magnets. After manipulation of the suture loop the magnets were often noted to be in the end-to-side configuration, preventing successful positioning of the suture loop.

**Figure 1:**
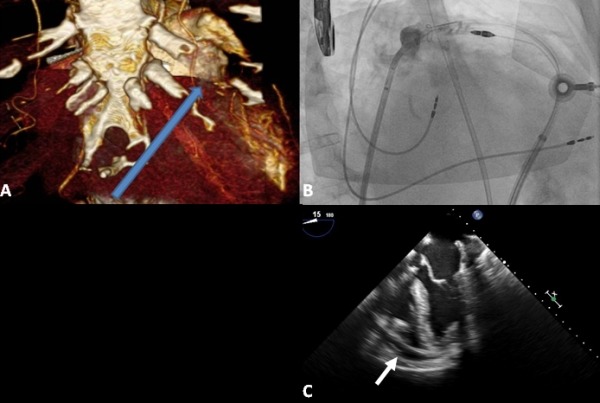
A. Reconstructed CT scan demonstrating anatomic relationship of the LAA and the ideal trajectory of the sub-xiphoid epicardial puncture. B. Contrast injection into the LAA os after closure of the Lariat suture showing no flow into the appendage. C. Intra-operative TEE prior to LAA closure demonstrates pericardial effusion.

During manipulation of the magnet-tipped guidewires, the patient became hypotensive to a systolic pressure of 55 mmHg and a new pericardial effusion was noticed on TEE (Figure 1). Approximately 200cc of blood was drained from the side arm of the epicardial sheath with some initial improvement in hemodynamics. The effusion reaccumulated and the blood pressure dropped. A pigtail catheter was emergently placed in the pericardial space. A total of 800cc was drained and autotransfused back to the patient. The patient's hemodynamic condition stabilized and the decision was made to continue the case. Hemostasis of the LAA would likely be achieved with successful application of the closure device.

With the pigtail catheter in place, a second subxiphoid epicardial access was obtained, directed more laterally from the first. The epicardial needle was directed towards the magnet tipped wire already in the LAA. The new orientation of the epicardial sheath was felt to improve access to the left atrial appendage.

**Figure 2:**
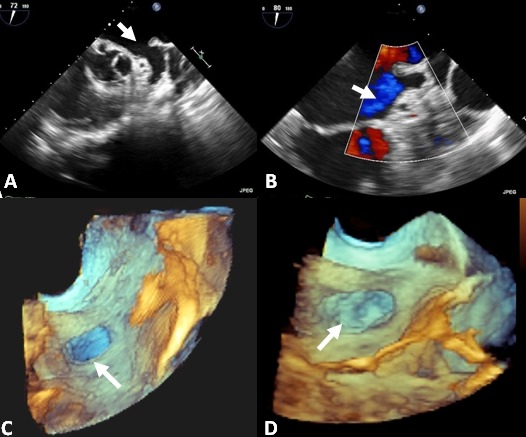
A. Pre-operative appearance of the LAA on 2D TEE. B. Post-operative TEE shows no color flow into the LAA. Bottom panels show 3D TEE images of the LAA pre- and post-operative (C and D, respectively)

The Lariat was advanced into the subxiphoid space through a second 13Fr sheath and easily tracked over the guidewire around the base of left atrial appendage. Appropriate position of the suture was confirmed with balloon inflation, atrial angiography, and color Doppler and 3D TEE (Figure 2).

The suture loop was successfully deployed and there was no further accumulation of the effusion. Intra-op TEE showed no residual flow in the appendage. The pigtail catheter was left in place. Protamine was administered to partially reverse anticoagulation.

Follow-up transthoracic echocardiography 12 hours later showed no pericardial effusion and the pigtail catheter was removed. The patient was later discharged home.

## Discussion

Percutaneous catheter-based occlusion of the LAA is a viable option in patients with limitations to oral anticoagulation therapy [1,2]. Devices available to accomplish this include the WATCHMAN (Aritech, Inc, Plymouth, MN), the Amplatzer device (Amplatzer Products, Plymouth, MN) and the Lariat (SentreHEART, Inc, Palo Alto CA).

The WATCHMAN device is a self expanding nitinol frame that is delivered percutaneously through a trans-septal approach. It is the only device currently undergoing FDA approval for stroke prevention in atrial fibrillation. However the Watchman LAA occlusion device requires 45 days of anticoagulation after implant. The Amplatzer device is now also being evaluated for LAA occlusion. Similar to the WATCHMAN device, initial anticoagulation is required for endothelialization [3].

The Lariat device is the only catheter-based non-intracardiac LAA closure device currently available. It is 510(k) USFDA approved for the soft-tissue approximation and/or ligation with a pre- tied polyester suture. Success is determined by angiography of the LAA and TEE color-flow Doppler. In contrast to occlusion devices that leave a device in contact with the bloodstream, anticoagulation is not needed after the procedure. Patients who cannot tolerate a minimum of 45 days of anticoagulation therapy are not candidates for current intracardiac LAA closure devices and are potential candidates for a Lariat suture closure device.

Initial experience with the Lariat procedure is now being reported. Early series in 3 centers show rate of acute or late effusion requiring intervention of 3.5 % (3 in 89 patients), 4% (1 in 25 patients) and 15% (3 in 20 patients) [4-6]. For comparison, the atrial fibrillation ablation tamponade rate is 1.2% in experienced hands and the epicardial VT significant effusion rate is 8% [7,8]. There has been one previously reported case of an LAA perforation during Lariat procedure with continuation of the procedure in order to not only ligate the LAA but also manage the complication. In that case there was no tamponade requiring drainage of the effusion, rather extravasation of contrast from the LAA angiogram was noticed on contrast injection [9].

An important component to the success of our procedure was our second more laterally oriented access to the epicardial space, directing it toward the appendage. This case emphasizes the importance of orientation of epicardial access. In a series of 85 patients who had successful LAA ligation, 17 of 85 required a second pericardial access attempt predominantly because the initial pericardial access attempt was too posterior or too medial [4]. The presence of the endocardial magnet-tipped wire already in the LAA offered directional guidance. Consideration should be given to the routine placement of the endocardial wire in the LAA before epicardial access in LARIAT atrial appendage exclusion procedures.

In our case, intra-operative development of tamponade lead us to consider reversing anticoagulation and aborting the procedure. However the onset of tamponade was delayed, well after the epicardial access, and did not occur until after extensive manipulation of the magnet-tip guidewires. This scenario favored the LAA as the most likely source. We recognized that closing the LAA would result in cessation of bleeding and we elected to continue the case as described.

This case highlights the importance of the lateral placement of the epicardial sheath and benefit obtained by having the endocardial wire in the LAA for guidance. It also illustrates how continuing with the Lariat snare suture exclusion of the LAA can treat the complication of LAA perforation.
